# Challenges in Diagnosing Dermoid Cyst in a Neurocognitive Patient

**DOI:** 10.1155/2024/5515676

**Published:** 2024-04-18

**Authors:** Alaa Safia, Rabie Shehadeh, Shlomo Merchavy

**Affiliations:** Department of Otolaryngology, Head and Neck Surgery, Ziv Medical Center, Safed, Israel

## Abstract

This case report presents a unique and challenging scenario involving the diagnosis and management of a sublingual dermoid cyst in a 12-year-old male with autism disorder. Dermoid cysts within the oral cavity are exceptionally rare entities, constituting less than 0.01% of all oral cavity cysts. In addition, their co-occurrence with neurocognitive disorders further complicates the diagnostic process. The patient's clinical presentation was marked by recurrent epistaxis and behavioral changes, which were compounded by his communication limitations due to autism disorder. A thorough physical examination revealed a sublingual mass causing tongue displacement. However, due to the patient's inability to effectively communicate symptoms, parental input played a pivotal role in constructing the clinical narrative. Imaging techniques, including computed tomography (CT) and magnetic resonance imaging (MRI), provided essential insights into the cyst's size, location, and structural characteristics. The successful integration of these modalities aided in achieving a definitive diagnosis. The subsequent intraoral surgical excision of the 6.5 cm cyst yielded a positive outcome, with an uneventful recovery and a six-month follow-up demonstrating no signs of recurrence. This report underscores the significance of multidisciplinary collaboration in navigating the complexities of diagnosing and managing rare oral lesions in patients with neurocognitive disorders. The confluence of two rare conditions necessitates a tailored approach that takes into account communication barriers and the patient's overall well-being. This case offers valuable insights to healthcare practitioners, highlighting the importance of leveraging advanced imaging technologies and adapting strategies to address the unique challenges posed by such cases. By presenting this exceptional clinical scenario, this report contributes to the medical community's understanding of nuanced diagnostic processes and patient-centered management techniques.

## 1. Introduction

Dermoid cysts are rare developmental lesions lined by epidermis-like epithelium and containing dermal adnexal structures. They are known to occur in various regions of the body, but their occurrence within the oral cavity is extremely uncommon, comprising less than 0.01% of all oral cavity cysts [1]. Among oral dermoid cysts, about seven percent are found in the floor of the mouth [2]. Typically presenting as slow-growing and asymptomatic masses, dermoid cysts are often diagnosed incidentally during routine examinations or investigations for unrelated symptoms [3]. However, in some cases, they can cause symptoms related to the compression of adjacent structures, resulting in difficulties in mastication, swallowing, and breathing [4].

Patients with neurocognitive disorders like autism face significant barriers in communicating symptoms related to medical conditions. Impaired verbal capacity and sensory issues can prevent accurate reporting of oral lesions [5]. Neurocognitive impairments in communication and social interaction may hinder the patient's ability to express symptoms related to oral lesions adequately. In this context, we report a case of a sublingual dermoid cyst in a 12-year-old male with autism disorder, highlighting the complexities in diagnosing and managing oral lesions in neurocognitive patients.

Imaging modalities, including ultrasound, computed tomography (CT), and magnetic resonance imaging (MRI), in conjunction with fine-needle aspiration (FNA), play a crucial role in facilitating accurate preoperative diagnosis. Surgical management remains the gold standard for treating dermoid cysts, and the surgical approach is determined based on the size and localization of the cyst. Favorable postoperative outcomes with low recurrence rates have been reported.

Recent advancements in MRI techniques, as discussed in Wang et al. [6], have significantly enhanced our ability to differentiate between benign and malignant oral lesions, a crucial step in the diagnosis of rare conditions like dermoid cysts. Furthermore, the evolution of surgical techniques, such as the three-dimensional exoscope elaborated in Ferlito et al. [7], offers promising outcomes in the treatment of these lesions, especially in patients with neurocognitive challenges.

By sharing our experience in managing this rare case of a dermoid cyst in a patient with autism disorder, we aim to highlight the diagnostic and management challenges encountered in a pediatric patient with both autism and a rare sublingual dermoid cyst through this case.

## 2. Case Presentation

A 12-year-old male with autism disorder presented with a 2-month history of nosebleeds, reduced oral intake, dysphagia, and decreased speech as reported by parents. He had no prior relevant medical or surgical history.

The patient's neurocognitive disorder posed challenges in gathering information directly from him, necessitating reliance on information provided by the parents.

**Figure 1:**
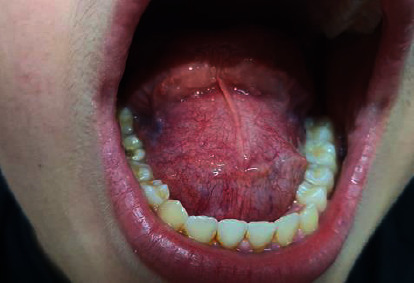
Clinical examination showing a sublingual mass with normal covering mucosa displacing the tongue superiorly.

Examination showed a nontender, sublingual cystic mass measuring 6.5 × 5.4 × 4.6 cm on imaging. It displaced the tongue superiorly with no mucosal abnormalities (Figure 1). No bluish discoloration, bleeding, or oozing was observed. The patient had no signs of dyspnea, dysarthria, or any previous oral cavity or neck surgeries. There were no palpable swellings in the submental space or neck lymphadenopathy. The patient did not report any weight loss or fever.

**Figure 2:**
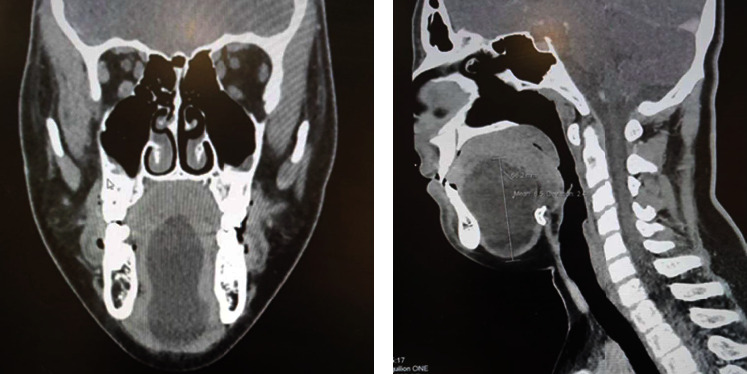
(a) Coronal and (b) sagittal CT scans revealing a homogeneous cystic structure measuring 6.5 cm in diameter with a delicate capsule without evidence of tissue component in the floor of the mouth, located centrally and anteriorly to the hyoid bone and below the tongue.

CT revealed a homogeneous, well-circumscribed cystic lesion inferior to the tongue measuring 6.5 cm in diameter in a central and anterior position to the hyoid bone, situated below the tongue, and encased by a delicate capsule (Figures 2(a) and 2(b)).

**Figure 3:**
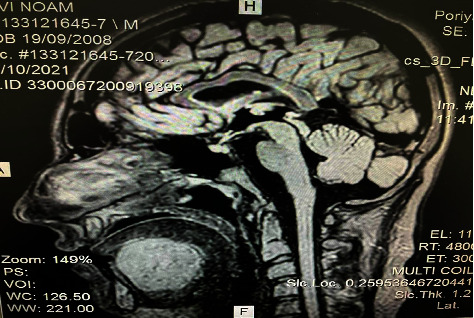
Sagittal MRI scan showing a homogeneous cystic lesion measuring 5.4 *∗* 3.1 *∗* 4.6 cm with a delicate capsule and uniform high-protein content liquid. Peripheral enhancement is observed after gadolinium injection in the sublingual space.

To obtain more detailed information, MRI demonstrated a thin-walled unilocular cyst with uniform high-protein content, peripheral enhancement was observed after gadolinium injection in the sublingual space (Figure 3).

**Figure 4:**
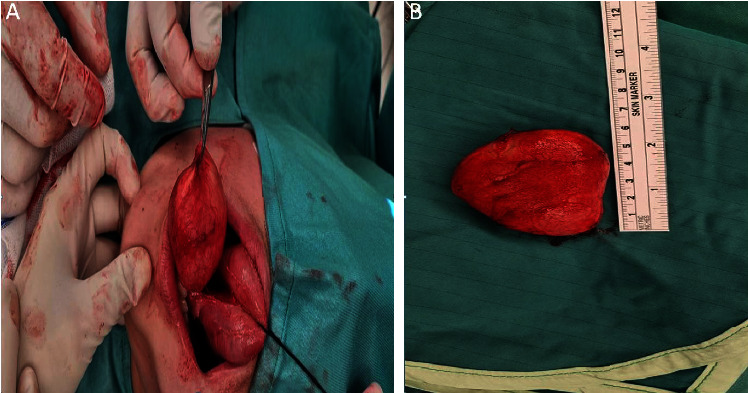
(a) Intraoral midline incision from the tongue base to the mouth floor and (b) view following removal of the cyst during surgery.

FNA was nondiagnostic, yielding only squamous cells. Due to the size and location of the cyst, surgical excision was undertaken through a midline sublingual incision, and the cyst was successfully excised (Figures 4(a) and 4(b)). The surgical site was closed primarily, and the patient had an uneventful recovery with no postoperative complications.

Histopathology showed benign squamous epithelium with hair follicles, confirming dermoid cyst diagnosis. No postoperative complications occurred over 6 months of follow-up.

## 3. Discussion

This case highlights the diagnostic challenges of evaluating rare oral lesions like dermoid cysts in patients with neurocognitive disorders. The patient's autism impaired his ability to communicate symptoms, necessitating greater reliance on parental input and physical exam findings.

Dermoid cysts have an estimated incidence of only 0.01% in the oral cavity, occurring anywhere along the midline from the floor of the mouth to the pharynx. Their rarity warrants consideration of other more common entities such as ranula, thyroglossal duct cyst, lymphatic malformations, and heterotopic gastrointestinal cysts in the differential diagnosis.

Dermoid cysts in the oral cavity can be either congenital or acquired. Congenital cysts result from the entrapment of ectodermal elements during the fusion of the first and second branchial arches within the first four weeks of pregnancy. Trauma and iatrogenic causes can lead to acquired dermoid cysts, as well as obstruction of sebaceous gland duct epithelial cells.

In this case, the sublingual location, patient age, cyst characteristics on CT/MRI, and absence of prior surgery pointed towards a congenital dermoid cyst despite the diagnostic limitations posed by the patient's autism.

The clinical presentation of oral cavity midline cysts varies, with some being slow-growing masses, while others may lead to dysphonia, dysphagia, and dyspnea due to tongue displacement. Dermoid cysts are typically diagnosed in the age group of 20–30 years.

Structural neuroimaging with CT and MRI were instrumental in delineating the size, extent, and internal composition of the lesion, guiding appropriate surgical planning. This underscores the value of advanced imaging in oral cystic lesions, particularly in patients with communication challenges.

Complete surgical excision remains the treatment of choice for dermoid cysts to prevent recurrence. The intraoral approach was suitable given the lesion's size and location. More extensive lesions may require extraoral techniques.

Postoperative follow-up is prudent as recurrence has been documented in up to 10% of the cases, attributed to incomplete removal or capsule rupture. No recurrence was observed over 6 months in this case.

## 4. Conclusion

Dermoid cysts in the oral cavity are exceedingly rare and require differential diagnosis from other oral pathologies. The presence of a neurocognitive disorder in the patient added complexity to the diagnosis and treatment process, emphasizing the importance of utilizing appropriate imaging techniques, such as ultrasound, CT, and MRI, in conjunction with fine-needle aspiration, for accurate diagnosis. Surgical removal remains the most effective management for these lesions, and prognosis is generally favorable, with low recurrence rates.
